# Effects of Postcuring Temperature on the Mechanical Properties and Biocompatibility of Three-Dimensional Printed Dental Resin Material

**DOI:** 10.3390/polym13081180

**Published:** 2021-04-07

**Authors:** Enkhjargal Bayarsaikhan, Jung-Hwa Lim, Seung-Ho Shin, Kyu-Hyung Park, Young-Bum Park, Jae-Hoon Lee, Jong-Eun Kim

**Affiliations:** 1 Department of Prosthodontics, Yonsei University College of Dentistry, Yonsei-ro 50-1, Seodaemun-gu, Seoul 03722, Korea; ejbayar@gmail.com (E.B.); drybpark@yuhs.ac (Y.-B.P.); jaehoon115@yuhs.ac (J.-H.L.); 2 Oral Research Science Center, BK21 FOUR Project, Department of Prosthodontics, College of Dentistry, Yonsei University, Yonsei-ro 50-1, Seodaemun-gu, Seoul 03722, Korea; erin850313@gmail.com (J.-H.L.); shin506@prostholabs.com (S.-H.S.); khyungpark@yuhs.ac (K.-H.P.)

**Keywords:** additive manufacturing, three-dimensional printing, rapid prototyping, CAD/CAM, postcuring, flexural strength, degree of conversion, cell viability, cytotoxicity, confocal laser scanning

## Abstract

Three-dimensional (3D) printing is an attractive technology in dentistry. Acrylic-based 3D printed resin parts have to undergo postcuring processes to enhance their mechanical and biological properties, such as UV-light and thermal polymerization. However, no previous studies have revealed how the postcuring temperature influences the biocompatibility of the produced parts. Therefore, we postprocessed 3D printed denture teeth resin under different postcuring temperatures (40, 60 and 80 °C) for different periods (15, 30, 60, 90 and 120 min), and evaluated their flexural properties, Vickers hardness, cell cytotoxicity, cell viability, and protein adsorption. In addition, confocal laser scanning was used to assess the condition of human gingival fibroblasts. It was found that increasing the postcuring temperature significantly improved the flexural strength and cell viability. The flexural strength and cell viability were 147.48 ± 5.82 MPa (mean ± standard deviation) and 89.51 ± 7.09%, respectively, in the group cured at 80 °C for 120 min, which were higher than the values in the 40 and 60 °C groups. The cell cytotoxicity increased in the 40 °C groups and for longer cultivation time. Confocal laser scanning revealed identifiable differences in the morphology of fibroblasts. This study has confirmed that the postcuring temperature influences the final mechanical and biological properties of 3D printed resin.

## 1. Introduction

The advent of computer-aided design (CAD) and computer-aided manufacturing (CAM) has brought a new modality to restorative dentistry, which has developed remarkably over the past 2 decades [1]. CAD/CAM has mainly utilized in subtractive manufacturing (SM), which is now being used routinely in various dental applications. By trimming a prefabricated block or disk, SM can be used to fabricate prostheses that are comparable with conventional prostheses with regard to biological properties, mechanical performance, and accuracy [2,3]. Although SM has some benefits such as standardized industrially fabricated materials that offer high quality finished products [4], SM technology has several drawbacks, such as greater waste of the raw material, wear of milling instruments, restrictions in the geometric details of the fabricated product in terms of undercuts and intaglio geometry, and only one unit being produced at a time [5]. Additive manufacturing (AM) is thus of greater interest than SM for the production of dental prostheses [6].

Noteworthy technological improvements in digital dentistry are now occurring in a method of additive manufacturing called three-dimensional (3D) printing due to its potential for rapid prototyping and the production of desired complex structures in a layer-by-layer manner from printable biomaterials [6,7]. Myriad photosensitive resins have been approved and used widely in restorative dentistry due to their good biocompatibility. The applications of 3D printing in dentistry include the production of dental casts, surgical guides, orthodontic and bite splints, temporary fixed dental prostheses, dentures, castable patterns and custom impression trays [8,9,10,11]. Moreover, numerous 3D printers, which utilize many AM methods such as stereolithography apparatus (SLA)/digital light projection (DLP), Multijet/Polyjet, selective laser sintering (SLS), and fused filament fabrication (FFF)/fused deposition modeling (FDM), are now commercially available for dental photosensitive resins [12]. In brief, SLA/DLP utilize UV lasers or UV LED light source to polymerize liquid photopolymers as the vat moves up or down, and thus generating 3D structures. Multijet/Polyjet printing, micrometer sized droplets of liquid photopolymer are dispensed from multiple print heads using 3-axis stages to build. SLS, on the other hand, the high temperature of the laser light is used to either sinter or weld specific regions of polymer powders. Lastly, FFF/FDM methods utilizes one or more heated nozzles that spatially distribute extruded polymer as a fine filament [12].

The type of 3D printer, materials, various printing and manufacturing parameters are known to influence the accuracy of printed objects [13,14]. There are biocompatible soft polymers such as silicone, polyurethane which can be fully cured during DLS/DLP printing process. Thus, they do not require postprocessing treatment [15]. However, postprocessing treatment such as thermal or UV-light curing is indicated for acrylic-based photosensitive resin in order to crosslink unreacted monomers and thereby complete the polymerization process after printing, which improves its final thermal and mechanical properties [16,17,18] depending on 3D printer type. Printable photosensitive resins consist essentially of monomers that are mostly based on (meth)acrylates [19], photo-initiators, and additives [20]. Once exposed to the appropriate light source, the polymerization involves free-radical reactions in which the material is transformed from a viscous to a rigid state. The terminal aliphatic C=C bonds are broken and converted to primary C–C covalent bonds between methacrylate monomers during the polymerization process [21]. The amount of polymerization is quantified as the degree of conversion (DC). Obtaining a higher DC usually provides better mechanical properties and biocompatibility [22,23], with residual monomer being decreased [24]. This is more critical for 3D printed dentures, temporary prostheses, and splints that are in contact with soft and hard tissues for much longer times than are implant surgical guides.

For these reasons, the postpolymerization process after printing process can play a crucial role in the final outcome. Previous studies have found that postpolymerization is highly dependent on the type of curing chamber, the frequency and intensity of the UV light, the exposure time, and the composition and color of the photosensitive resin, which can potentially affect the mechanical strength and biocompatibility of the 3D printed resin [25], as well as its accuracy [26]. Salmoria et al. revealed that thermal curing of the resin used in an SLA 3D printer can reduce the cure heterogeneity and anisotropy [27]. It was also demonstrated that the postcuring time and temperature influenced the DC [26]. Kumar et al. performed postpolymerization at curing temperatures from 80 to 140 °C for different postcuring times, which revealed that postcuring at 140 °C for 6 hours (h) resulted in better thermal and mechanical properties [28]. Furthermore, 3D printed resins when applied for provisional fixed dental prostheses and dentures must withstand occlusal forces, thus ensuring sufficient mechanical properties for clinical application is important. Reymus et al. investigated fracture load of 3D printed provisional three-unit bridge and concluded that postcuring affects mechanical properties of 3D printed resin material [29]. However, no previous study has investigated the biocompatibility of the final 3D printed dental resin products postpolymerized under various curing temperatures. Moreover, mechanical properties of the final 3D printed dental resin are not extensively evaluated in previous studies. 

Hence, in order to gain a better understanding of the effects of various postpolymerization conditions on the mechanical strength and biocompatibility of 3D printed resin specimens, we investigated how postcuring parameters influence the flexural strength, DC, and biocompatibility of 3D SLA-printed denture teeth resin. The null hypothesis was that postcuring parameters would significantly affect the mechanical properties, DC, and biological properties of a 3D printed object.

## 2. Materials and Methods

### 2.1. 3D CAD Design and 3D Printing

Specimens were designed using CAD software (Meshmixer, Autodesk, San Rafael, CA, USA) prior to printing. For the mechanical-property tests, a bar was designed with a length of 25 mm, a width of 2 mm, and a thickness of 2 mm according to the ISO 10477 standard. The surface hardness, DC, and biocompatibility test were investigated using a disk with a diameter of 9 mm and a thickness of 2 mm. The specimen designs were saved as STL (Standard Tessellation Language) files and exported into a 3D printing software program (PreForm Software, Formlabs, Somerville, MA, USA). The bar and disk specimens were prepared in 120° and 0° orientations, respectively, which were printed in three dimensions using commercially available PMMA resin (Denture teeth resin A2, Formlabs) with a layer thickness of 50 μm using a desktop SLA 3D printer (Form 3, Formlabs). The 3D printer used in this study was powered by Low Force Stereolithography (LFS)™ using 405 nm UV light with a laser power of 250 mW. 

### 2.2. UV-Light Postcuring Protocol for 3D Printed Specimens

**Figure 1:**
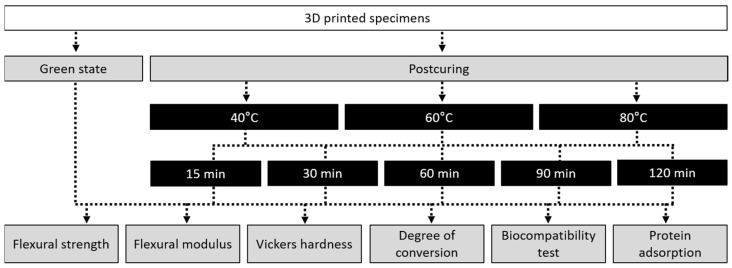
Workflow of the overall experimental process, showing the postcuring conditions and types of experiment.

After printing, the support structures were removed and the residual resin monomer on the 3D printed specimens was cleaned with 90% isopropanol using a 3D printing washer (Twin Tornado, MEDIFIVE, Incheon, Korea) for 10 min. The specimens were then postcured in a UV-light polymerization chamber (FormCure, Formlabs) using a 405 nm light source (13 multidirectional LEDs) and capable of heating up to 80 °C. The printed specimens were postcured at temperatures of 40, 60 and 80 °C for 15, 30, 60, 90 and 120 min. The specimens in the green state (GS) (without postcuring) were used as a control group (Figure 1).

### 2.3. Flexural Strength and Flexural Modulus Test

Fifteen bar specimens per group (*n* = 15) were stored in at 37 °C for 24 h according to the ISO 10477 standard before testing. They were loaded using a universal testing machine (Model 3366, Instron Corporation, Norwood, MA, USA) with a crosshead speed of 1 mm/min. Each specimen was placed on two rounded supports with a span length of 20 mm parallel to each other. It was then uniaxially loaded in the middle until the specimen failed, and the maximum force before fracturing was recorded in newtons.

**Figure 2:**
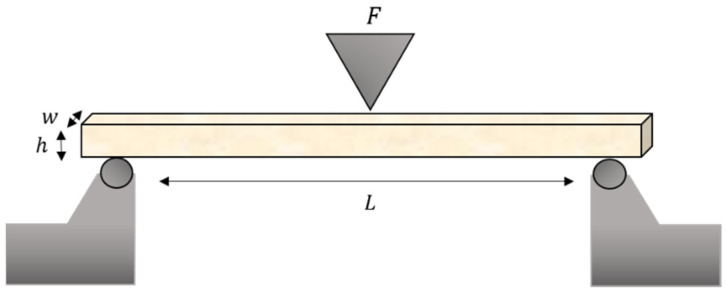
Schematics of the test setup for ISO 10447 flexural strength.

The flexural strength (*σ*) in megapascals and the flexural modulus (*E*) in gigapascals were calculated as follows [13]:

(1) $$\sigma =\frac{3FL}{2wh^{2}}$$

(2) $$E=\frac{FL^{3}}{4wh^{3}d}$$

where *F* is the load at a selected point of the elastic region of the stress–strain plot, *L* is the span length between the supports in millimeters, *w* is the width of the specimen in millimeters, *h* is the height of the specimen in millimeters, and *d* is the deflection of the specimen in millimeters (Figure 2).

### 2.4. Vickers Hardness Test

Disks (*n* = 5) were polished with increasing fineness using SiC paper (2000 and 4000 grit, sequentially) and stored in a 37 °C incubator for 24 h. A Micro Vickers hardness tester (HMV-G31ST, Shimadzu Co. Ltd., Kyoto, Japan) was used to determine specimen hardness by applying a force of 200 g (1.96 N) for 15 s [30]; an average value was calculated from five different locations on each specimen.

### 2.5. Degree of Conversion

Printed disks were polished with SiC paper (4000 grit) and stored in ambient conditions for 24 h. The spectrum of the postcured specimen and that of the unpolymerized resin were obtained using a Fourier transform infrared spectrometer (Nicolet IS10, Thermo Scientific, Thermo Electron Scientific Instruments, Madison, WI, USA). The absorbance was measured under the following conditions: 16 scans, 4 cm^−1^ resolution, and wavelengths from 750 to 4000^−1^ cm. For measuring the unpolymerized state of the material, a drop of monomer resin was applied on a microscope slide and measured. Three measurements were performed on the top surface of each disk, and the average of these scans was analyzed. The peak areas for aliphatic and aromatic C=C double bonds at 1638 and 1608 cm^−1^, respectively, were analyzed [25,31]. The DC was calculated as follows:

(3) $$DC\;\left(\%\right)=\;\left[1-\;\frac{1638\;{\mathrm{cm}}^{-1}/1608\;{\mathrm{cm}}^{-1}{}_{Peak\;height\;\left(cured\right)}}{1638\;{\mathrm{cm}}^{-1}/1608\;{\mathrm{cm}}^{-1}{}_{Peak\;height\;\left(monomer\right)}}\right]\;\times 100$$

Mean ± standard deviation values were calculated from the measurements made at the three positions.

### 2.6. Biocompatibility Test

#### 2.6.1. In Vitro Cell Culture and Cell Line

Primary human gingival fibroblasts (HGFs; PCS-201-018, ATCC, Manassas, VA, USA) were cultured in a cell culture dish with Dulbecco’s modified Eagle’s medium (DMEM, WelGene, Daegu, Korea) supplemented with penicillin/streptomycin (Penicillin-Streptomycin, 100X, WelGene), MEM nonessential amino acid solution (MEM Non-Essential Amino Acid Solution, 100X, WelGene), and 10% fetal bovine serum (FBS, Thermo Scientific, Waltham, MA, USA) at 37 °C in 5% CO_2_, 95% air atmosphere, and 100% relative humidity. The cell culture medium was changed every 2–3 days. When the cells reached 85–90% confluency, they were treated with trypsin-ethylenediaminetetraacetic acid solution (Trypsin-EDTA, 1X, WelGene) and seeded into 48-well plates at a density of 5 × 10^4^ cells/well for each specimen. Cells were allowed to attach for 24, 48 and 72 h at 37 °C in 5% CO_2_ before cell viability and cytotoxicity assays.

#### 2.6.2. Cell Viability and Cytotoxicity Assays

Cell viability was evaluated using a CELLOMAX™ viability kit based on the tetrazolium salt (2-(2-methoxy-4-nitrophenyl)-3-(4-nitrophenyl)-5-(2,4-disulfophenyl)-2H-tetrazolium and monosodium salt [WST-8]; Precaregene, Hanam, Kyungido, Korea). After 24, 48 and 72 h of incubation, 50 μL of CELLOMAX™ solution (CELLOMAX™ viability assay kit, Precaregene) was added to the 48-well plate with the specimen and incubated for 90 min at 37 °C according to manufacturer’s instructions. Subsequently, 100 μL aliquots of the media from each well were transferred to the 96-well plate and their absorbance was recorded using a microplate reader (VERSA max, Molecular Devices, Sunnyvale, CA, USA) at a wavelength of 450 nm. The percentage of cell viability was calculated as follows:

(4) $$Cell\;viability\;\left(\%\right)=\;\left(\frac{{\mathrm{OD}}_{test\;sample}-{\mathrm{OD}}_{blank}}{{\mathrm{OD}}_{control}-{\mathrm{OD}}_{blank}}\right)\;\times 100$$

The cytotoxicity of the 3D printed resin on HGFs was measured using a lactate dehydrogenase (LDH) release assay (Quanti-LDH™ Cytotoxicity Assay Kit, BIOMAX, Seoul, Korea) according to the manufacturer’s protocol. After 24, 48 and 72 h, 20 μL of the culture medium were collected and centrifugated at 7000 rpm for 3 min. Then, 10 μL of the obtained supernatant was transferred to a new 96-well plate, to which 100 μL of the LDH substrate mixture was added. After 30 min of incubation at room temperature, the absorbance of the resultant solution was measured at 450 nm. For the cell cytotoxicity assay, the media were not changed until 72 h. The percentage of cytotoxicity was calculated as follows:

(5) $$Cytotoxicity\;\left(\%\right)=\;\left[\frac{\left({\mathrm{OD}}_{cells\;with\;3D\;printed\;resin}-{\mathrm{OD}}_{background\;control}\right)-{\mathrm{OD}}_{low\;control}}{{\mathrm{OD}}_{high\;control}-{\mathrm{OD}}_{low\;control}}\right]\;\times 100$$

#### 2.6.3. Cytoskeleton Staining and Confocal Laser Scanning Microscopy

After a cultivation time of 24 h, specimens were washed three times with PBS and the cells were fixed with 4% paraformaldehyde in PBS for 15 min at room temperature. The specimens were then washed again three times, permeabilized with 0.1% Triton™ X-100 in PBS for 15 min, washed once again three times with PBS. For the cytoskeleton quantification, phalloidin (Alexa Flour 488^®^ Phalloidin, Invitrogen, Thermo Scientific) was used to fluorescently stain the cytoskeleton via its binding to β-actin, in accordance with manufacturer’s instructions. The specimens were subsequently mounted in antifade mounting media (VECTASHIELD, Vector Laboratories, Burlingame, CA, USA) and scanned using confocal laser scanning microscopy (CLSM; LSM 700, Carl Zeiss Microscopy, Jena, Germany). Co-expression was confirmed in Z-stacked serially captured images.

### 2.7. Protein Adsorption Assay

Three specimens per group were placed into 48-well plate for the protein adsorption. A 1-mg/mL protein stock solution was prepared from bovine serum albumin (BSA; Sigma-Aldrich, St. Louis, MO, USA). Each 3D printed disk was immersed into 500 μL of the protein stock solution. After 24 h of incubation under sterile humidity conditions at 37 °C in 5% CO_2_, any unadhered protein was removed by washing three times with PBS. Diluted BSA standards and bicinchoninic acid (BCA) working reagent were prepared according to the manufacturer’s protocol (Pierce™ BCA Protein Assay Kit, Thermo Fisher Scientific, Rockford, IL, USA). A total of 1 mL micro BCA working reagent was used for the colorimetric detection and quantitation of total protein, followed by incubation at 37 °C in 5% CO_2_ for 30 min. Thereafter, 100 μL aliquots were transferred to 96-well plate and the absorbance was recorded using a microplate reader (VERSA max, Molecular Devices) at 562 nm. Standard curves were prepared using the BSA standard. The protein concentration was used to calculate the amount of protein adsorbed on the surface of 3D printed disks.

### 2.8. Statistical Analysis

Data for the flexural strength, flexural modulus, Vickers hardness test, DC, and protein adsorption were analyzed using one-way ANOVA and two-way ANOVA followed by the Bonferroni pairwise comparison and Tukey multiple-comparisons test. The data from the cell viability and cytotoxicity assays and for protein adsorption were analyzed using three-way ANOVA and the *t*-test using standard statistical software (version 25.0, SPSS Statistics, IBM, Armonk, NY, USA) (α < 0.05).

## 3. Results

### 3.1. Flexural Strength

**Figure 3:**
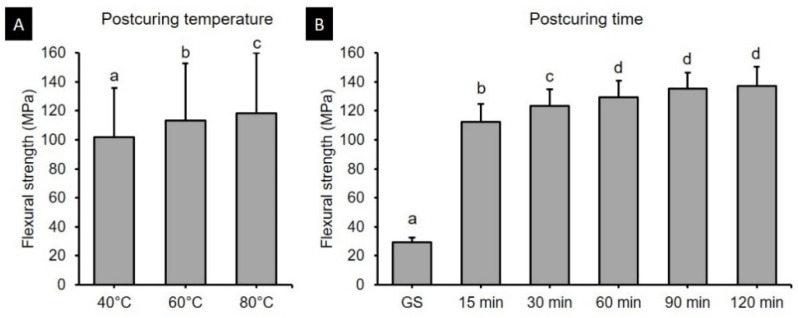
Results from two-way ANOVA. Flexural strength according to postcuring (**A**) temperature and (**B**) time. Data are mean and standard-deviation values of 3D printed specimens. Different lowercase letters indicate a significant difference.

**Figure 4:**
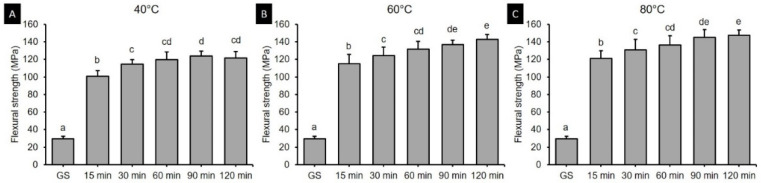
Results from one-way ANOVA. Flexural strength according to postcuring time for each postcuring temperature. (**A**) 40 °C (**B**) 60 °C and (**C**) 80 °C. Data are mean and standard-deviation values of 3D printed specimens. Different lowercase letters indicate a significant difference in the same group.

Two-way ANOVA was performed to verify the effects of postcuring temperature and time on the flexural strength. It was found that both the postcuring temperature (*F* = 113.416, *p* < 0.001) and the postcuring time (*F* = 1297.688, *p* < 0.001) significantly affected the flexural strength (Figure 3). There was also a significant interaction effect between the postcuring temperature and time (*F* = 5.876, *p* < 0.001). Overall, the flexural strength did not significantly improve more than 60 min of postcuring time (129.12–137.3 MPa) (Figure 3B). Figure 4 shows the flexural strength in groups with different postcuring times for each postcuring temperature. The flexural strength varied significantly with the curing time and temperature, being highest for 120 min at 80 °C (147.48 ± 5.82 MPa) and 90 min at 80 °C (145.13 ± 8.73 MPa) (Figure 4C). For the groups with 15 min of postcuring time, the flexural strength was significantly lower for postcuring temperatures of 40 °C (100.70 ± 6.65 MPa), 60 °C (115.00 ± 10.55 MPa), and 80 °C (121.35 ± 8.70 MPa) than the other postcuring time.

### 3.2. Flexural Modulus

**Figure 5:**
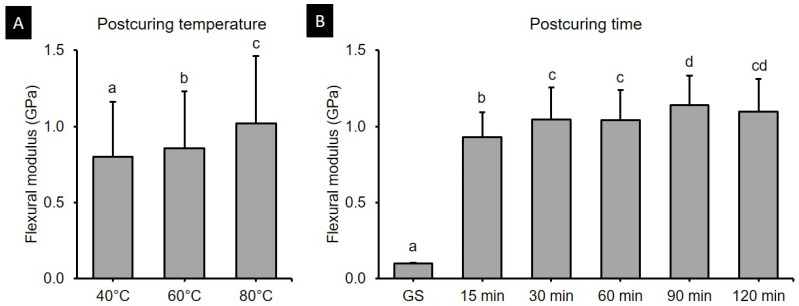
Results from two-way ANOVA. Flexural modulus data according to (**A**) postcuring temperature and (**B**) time. Data are mean and standard-deviation values. Different lowercase letters indicate a significant difference.

**Figure 6:**
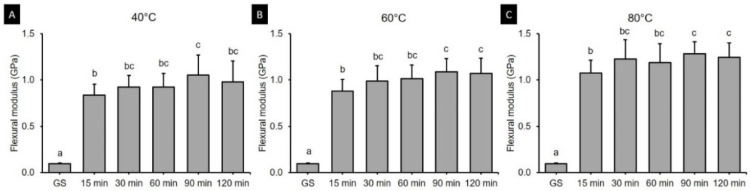
Results from one-way ANOVA. Flexural modulus according to postcuring time for each postcuring temperature. (**A**) 40 °C (**B**) 60 °C and (**C**) 80 °C. Data are mean and standard-deviation values. Different lowercase letters indicate a significant difference in the same group.

The two-way ANOVA (Figure 5) revealed that the postcuring temperature (*F* = 50.191, *p* < 0.001) and the postcuring time (*F* = 309.135, *p* < 0.001) significantly affected the flexural modulus. A significant interaction effect (*F* = 2.226, *p* < 0.05) was found between postcuring temperature and time. The flexural strength was significantly higher for a postcuring temperature of 80 °C (1.02 ± 0.45 GPa, *p* < 0.0001) than for those of 40 °C (0.80 ± 0.36 GPa) and 60 °C (0.86 ± 0.37 GPa (Figure 5A). Specimens in the GS group did not break during three-point bending tests, with the maximum exerted load being significantly lower among all groups (0.10 ± 0.01 GPa) (Figure 5B). The flexural modulus for each postcuring temperature is presented in Figure 6. The flexural modulus of the 3D printed resin used in this study increased with the postcuring time and temperature, and reached a plateau after 90 min of postcuring. The flexural modulus was highest for 90 min at 80 °C (1.28 ± 0.13 GPa) and for 120 min at 80 °C (1.24 ± 0.16 GPa), and lowest for 15 min at 40 °C (0.84 ± 0.12 GPa).

### 3.3. Vickers Hardness Test

**Figure 7:**
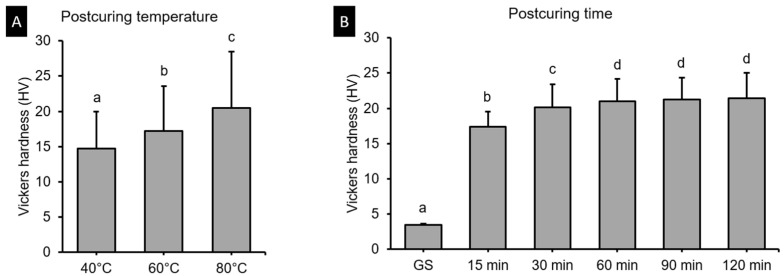
Results from two-way ANOVA. Vickers hardness data according to postcuring (**A**) temperature and (**B**) time. Data are mean and standard-deviation values. Different lowercase letters indicate a significant difference.

**Figure 8:**
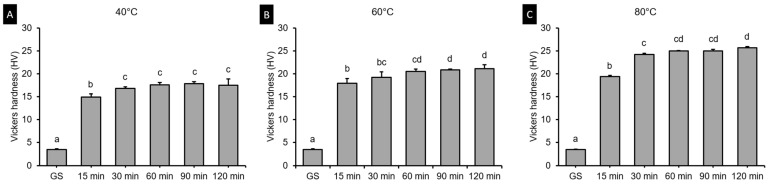
Results from one-way ANOVA. Vickers hardness according to postcuring time for each postcuring temperature. (**A**) 40 °C (**B**) 60 °C and (**C**) 80 °C. Data are mean and standard-deviation values. Different lowercase letters indicate a significant difference in the same group.

Figure 7 shows result of two-ANOVA. The postcuring temperature (*F* = 563.338, *p* < 0.001) and the postcuring time (*F* = 1653.244, *p* < 0.001) significantly affected the Vickers hardness. The Vickers hardness was 3.47 in the green state, and it increased to 14.89–17.51 in the 40 °C groups (Figure 8A), and to 17.95–21.16 in the 60 °C groups (Figure 8B), and to 19.4–25.7 in the 80 °C groups (Figure 8C).

### 3.4. Degree of Conversion

**Figure 9:**
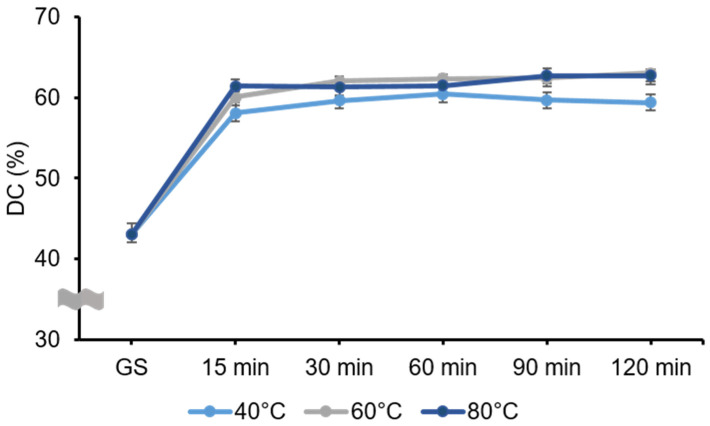
Degree of conversion (DC) of the 3D printed resin specimens that were postcured under different postcuring conditions. DC gradually increased after 15 min of postcuring for all temperatures. Data are mean and standard-deviation values.

The results for the DC are shown in the Figure 9. The two-way ANOVA revealed that the postcuring temperature (*F* = 97.507, *p* < 0.001) and the postcuring time (*F* = 1856.26, *p* < 0.001) significantly affected the DC. There was also a significant interaction effect between curing temperature and time (*F* = 7.475, *p* < 0.001). The DC was significantly higher in the 60 °C (58.80 ± 7.22%) and 80 °C (58.76 ± 7.17%) groups than in the 40 °C group (56.70 ± 6.29%). Relative to GS specimens, the polymerization rate increased dramatically in the postcuring temperature, and was highest for 120 min at 60 °C (63.37 ± 0.42%) and lowest for 15 min at 40 °C (58.07 ± 1.28%) among the postcuring groups. However, after 60 min of postcuring, the polymerization rate did not vary significantly (*p* = 0.837) with the postcuring temperature.

### 3.5. Biocompatibility Test

#### 3.5.1. Cell Viability Assay

**Figure 10:**
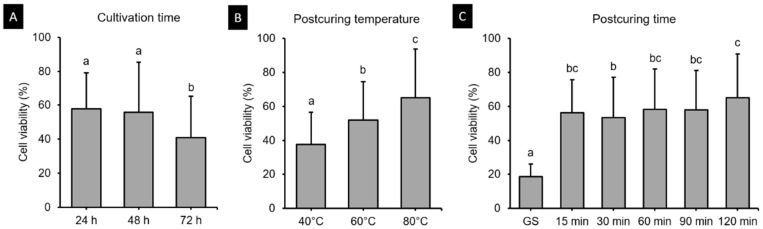
Results from three-way ANOVA. Cell viability data are presented according to (**A**) cultivation time, (**B**) postcuring temperature, and (**C**) postcuring time. Mean and standard-deviation values are shown. Different lowercase letters indicate a significant difference.

**Figure 11:**
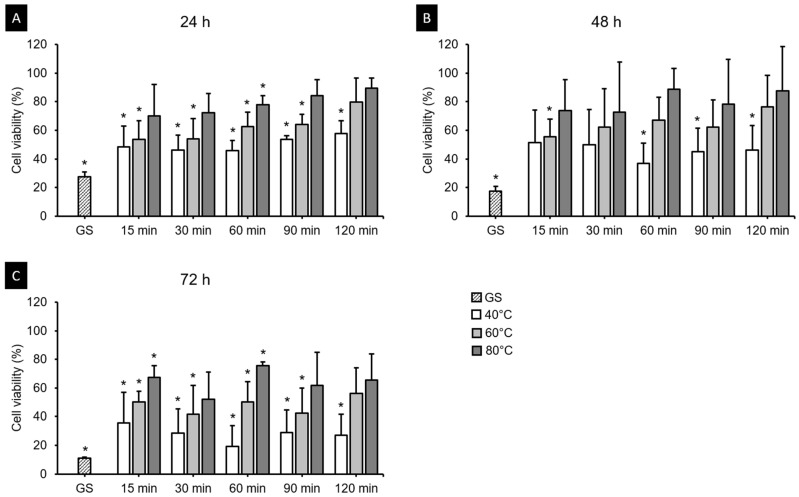
Results from cell viability assays. Cell viability increased with the postcuring temperature after (**A**) 24 h, (**B**) 48 h and (**C**) 72 h of experimentation. The cell viability gradually increased with the postcuring temperature and time. Mean and standard-deviation values are shown. Asterisk indicates a significant difference from the PC in the *t*-test.

The HGFs were cultured on 3D printed specimens, and after 24, 48 and 72 h, cell viability assays demonstrated different results according to postcuring temperature and time (Figure 10). The three-way ANOVA revealed that cell viability differed significantly with the cultivation time (*F* = 36.204, *p* < 0.001), postcuring temperature (*F* = 79.775, *p* < 0.001), and postcuring time (*F* = 57.386, *p* < 0.001). There was a significant interaction effect between cultivation time, postcuring temperature, and postcuring time (*F* = 1.580, *p* < 0.05). Cell viability was lowest in the GS group, and increased continuously with the postcuring time (Figure 10C). After 24 h, the cell viability was highest for 120 min at 80 °C (89.51 ± 7.09%) and lowest for 15 min at 40 °C (48.53 ± 14.58%) (Figure 11A). At 48 and 72 h, the cell viability was higher in the 80 °C group (69.73 ± 33.96% and 55.58 ± 25.36%, respectively) than in the 40 and 60 °C groups (Figure 11B,C). According to *t*-tests, at 48 h of cell culturing, the cell viability did not differ significantly between the 80 °C group and the positive control (PC) (*p* > 0.05, Figure 11B), which refers to natural cell viability without a 3D printed specimen during the experiment.

#### 3.5.2. Cell Cytotoxicity Assay

**Figure 12:**
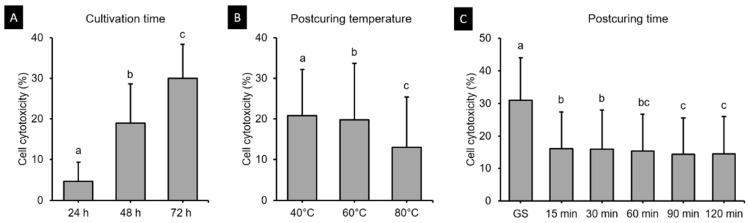
Results from three-way ANOVA. Cell cytotoxicity according to (**A**) cultivation time, (**B**) postcuring temperature, and (**C**) postcuring time. Mean and standard-deviation values are shown. Different lowercase letters indicate a significant difference.

**Figure 13:**
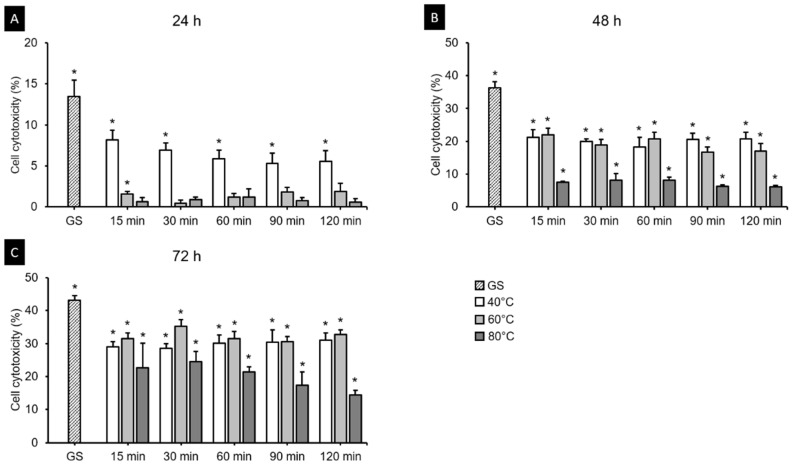
Results from cell cytotoxicity assays after (**A**) 24 h, (**B**) 48 h and (**C**) 72 h. The cell cytotoxicity gradually increased with the cultivation time but decreased with the postcuring temperature. Mean and standard-deviation values are shown. Asterisk indicates a significant difference from the positive control (PC) in the *t*-test.

Figure 12 shows the cell cytotoxicity of the 3D printed resin samples according to cultivation time, postcuring temperature, and postcuring time. The three-way ANOVA revealed that cell cytotoxicity differed significantly with the cultivation time (*F* = 2920.851, *p* < 0.001), postcuring temperature (*F* = 327.132, *p* < 0.001), and postcuring time (*F* = 376.927, *p* < 0.001). There was also a significant interaction effect between cultivation time, postcuring temperature, and postcuring time (*F* = 13.687, *p* < 0.001). Overall, the cell cytotoxicity was significantly lower for a postcuring time of 90 min (14.41 ± 11.17%) than for those of 15 min (16.02 ± 11.46%, *p* = 0.01) and 30 min (15.96 ± 11.99%, *p* = 0.016), but did not differ significantly from those in the 60 min group (15.42 ± 11.31%, *p* = 0.268) or the 120 min group (14.44 ± 11.55%, *p* = 1.000) (Figure 12C). Figure 13 shows the results from the LDH assays of HGFs cultured in the 3D printed specimens on different cultivation times. The *t*-tests showed that the cytotoxicity at 24 h was lower in the 80 °C (2.93 ± 4.89%) and 60 °C (3.41 ± 4.70%) groups than in the 40 °C group (7.55 ± 3.11%), and that it did not differ significantly from that in the PC (*p* > 0.05) except for 15 min at 60 °C (*p* = 0.026) (Figure 13A). However, from 48 h the cytotoxicity increased in all of the groups and differed significantly from that in the PC (*p* < 0.05) (Figure 13B). The cytotoxicity was higher at 72 h than 48 h, but the trend was similar to that at 48 h (Figure 13C). The cytotoxicity was lower for 120 min at 80 °C (7.04 ± 5.97%) throughout all cultivation times. We found that the cytotoxicity decreased as the postcuring temperature increases. 

#### 3.5.3. CLSM Analysis

**Figure 14:**
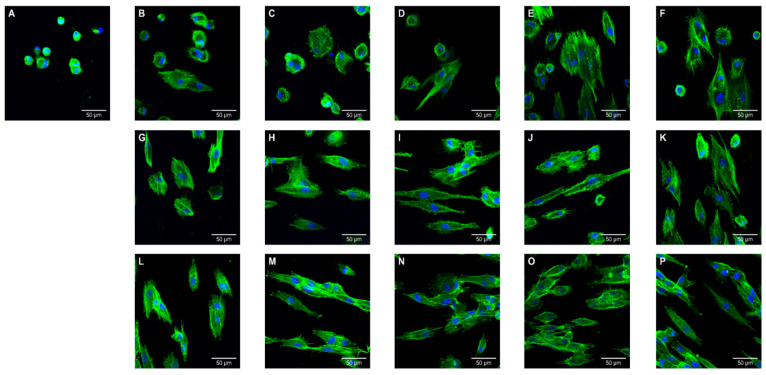
Confocal laser scanning microscopy (CLSM) images of human gingival fibroblasts cultures (5 × 10^4^ cells/mL) on 3D printed specimens postcured at various temperature and times after 24 h of incubation: (**A**) GS, (**B**) 40 °C for 15 min, (**C**) 40 °C for 30 min, (**D**) 40 °C for 60 min, (**E**) 40 °C for 90 min, (**F**) 40 °C for 120 min, (**G**) 60 °C for 15 min, (**H**) 60 °C for 30 min, (**I**) 60 °C for 60 min, (**J**) 60 °C for 90 min, (**K**) 60 °C for 120 min, (**L**) 80 °C for 15 min, (**M**) 80 °C for 30 min, (**N**) 80 °C for 60 min, (**O**) 80 °C for 90 min, and (**P**) 80 °C for 120 min.

CLSM observations of cell morphology on the surface of specimens are shown in Figure 14. The GS group showed relatively a small number of adhered cells and poor morphology. Differences of morphology and size became more obvious as the postcuring temperature increased, which large numbers of multinucleate cells (stained blue), and well-stretched cytoplasm and well-grown filopodias (stained green). All 40 °C groups exhibited fibroblasts with small and round morphology, and some cells in the specimens processed at 40 °C for 90 min and 40 °C for 120 min remaining round with few filopodias being produced at the cell edges. For 60 °C groups, most cells showed an elongated shape or dendritic morphology, although a very small number of the cells remained round. There were noticeable differences between the 40 and 60 °C groups. For all 80 °C groups, many more cell–cell contacts were detected on the surface of the specimens compared with the 40 and 60°C groups. Increasing the postcuring temperature and time resulted in more cells appearing stretched and in contact with neighboring cells.

### 3.6. Protein Adsorption Assay

**Figure 15:**
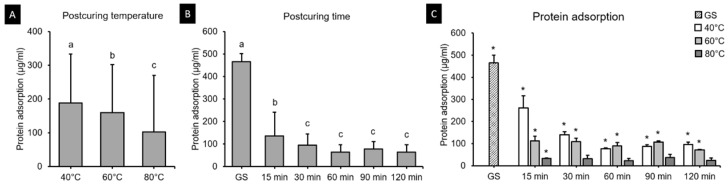
Results from protein adsorption assays. Mean and standard-deviation values are shown. (**A**) Two-way ANOVA showed that the protein adsorption decreased with postcuring temperature and (**B)** did not differ significantly after 30 min of postcuring time. Different lowercase letters indicate a significant difference. (**C**) The protein adsorption for 40, 60 and 80 °C groups. Asterisk indicates a significant difference from the PC in the *t*-tests.

Protein adsorption onto the 3D printed resin surfaces is shown in Figure 15. The two-way ANOVA test revealed a significant interaction effect between postcuring temperature and time (*F* = 5528.854, *p* < 0.001) (Figure 12A,B). The protein adsorption differed significantly for 40 °C (77.67–262.67 µg), 60 °C (72.67–112.13 µg), and 80 °C (22.83–36.33 µg) groups. According to *t*-tests, protein adsorption was significantly lower for 30, 60, 90 and 120 min at 80 °C, and did not differ significantly from that for the PC (*p* > 0.05) (Figure 12C).

## 4. Discussion

In this study, the effects of different combinations of postcuring temperatures and times were evaluated. From the above results testing the two key parameters of postcuring temperature and time, we observed significant influence of change in temperature on the 3D printed resin properties whereas for time differences were not discernible. Therefore, the null hypothesis was partially accepted.

The present study found that the flexural strength of 3D printed denture teeth resin increased significantly with the postcuring temperature. Postcuring temperature improves the anisotropy that provides more-homogeneous curing for 3D printed specimens, which results in better mechanical properties. In addition, a higher temperature would accelerate polymerization by enhancing the diffusion of free radicals and thereby increasing the potential for them to react throughout the specimen [32,33]. In the present study, the flexural strength of the 3D printed specimens increased gradually until 120 min of postcuring time, although there was no significant difference for postcuring times of longer than 60 min (Figure 3B). Another study also found that the flexural strength increased with the postcuring time [34]. In our study the flexural strength increased abruptly compared with the GS group after 15 min of postcuring. However, another previous study found no significant differences in flexural strength between their GS group and the group with 15 min of postcuring [25]. Moreover, none of the specimens in the present GS group fractured during the three-point bending test, which demonstrates the presence of flow plasticity. The printing parameters of the 3D printer also appear to affect the mechanical properties of printed specimens in their GS. In terms of the flexural modulus, the tendency was similar to that for the flexural strength for both postcuring temperature and time. The flexural modulus of 3D printed specimens increased significantly with the postcuring temperature. This could be explained by the energy received by the system increasing with the temperature so as to activate the monomers to further crosslink [28]. It was observed that a higher postcuring temperature resulted in a smaller displacement being required for fracturing the sample. Jindal et al. investigated the mechanical properties of 3D printed clear aligners under different postcuring conditions and found that good flexural properties could be achieved by using a higher temperature (80 °C for 10 or 20 min) with a shorter postcuring time compared with lower temperatures (60 °C for 20 min or 40 °C for 20 min) [10]. These findings are consistent with those of our study.

The Vickers hardness test demonstrated significant difference according to postcuring temperature. Higher postcuring temperature improves surface hardness significantly. Overall, Vickers hardness of the 3D printed specimens increased gradually until 120 min of postcuring time. However, there was no significant difference for postcuring times of longer than 60 min. This trend was similar with the result of flexural properties.

The DC is strongly influenced by several factors, including the exposure time, power density, and wavelength of the light source, and the distribution, material composition, and final characteristics of the resin [35]. Previous studies have also found that mechanical and chemical properties are dependent on the DC, and thus may play crucial roles in determining the ultimate success of the restoration [36,37]. In our study, no significant difference was found in the DC between groups postcured at 60 and 80 °C. Katheng et al. evaluated the DC of 3D printed resin postcured at 40, 60 and 80 °C, and found that it was highest for 30 min at 60 °C (90.92 ± 3.18%) [26]. In that study, the DC of resin that was postcured for 15 and 30 min at 60 °C differed significantly from that postcured for 15 min at 40 °C (79.42 ± 10.02%), 30 min at 40 °C (72.19 ± 7.97%), 15 min at 80 °C (73.94 ± 9.51%), and 30 min at 80 °C (75.72 ± 6.92%) [26]. The pattern of these results is similar to that found in our study.

A particularly interesting finding of the present study was of no specific improvement in the DC for the 80 °C groups relative to the 60 °C groups (Table S1). This might indicate that an oxygen inhibition layer forms on the surface of the resin due to oxygen molecules attaching to free radicals in the polymerization process, with this layer inhibiting the cure process on the surface [38,39]. Such an inhibition process would be limited to the most-superficial layer of the surface [33]. During the postcuring process, UV light polymerizes not only the surface of the resin but also penetrates a certain depth [40], and the uniformity of the DC throughout the specimen has a decisive impact on both its biocompatibility and mechanical properties. Furthermore, the maximum penetration of the spectrometer was 10 µm, and so only the absorption of the superficial layer of the resin could be measured [41]. For this reason, the present study was limited to measuring the DC on the specimen’s outer surface. 

Figure 7B indicates that the postcuring temperature had a significant impact on the cell viability. Cell viability tended to increase gradually with the postcuring time, but there were no significant changes after 60 min. A similar result was obtained in a previous study [25]. At 48 h, the 3D printed specimens cured at 60 and 80 °C did not differ significantly from the PC (Figure 8B), whereas cell viability was significantly decreased at 72 h compared with 24 and 48 h. One possible explanation is that unpolymerized monomer in the deeper layer of the resin leached out to induce a cytotoxic effect [41]. The cytotoxicity of a material is determined mainly by the degree of polymerization on its surface, which is the summation of the degree of polymerization induced during 3D printing plus the further polymerization induced by postpolymerization [42]. As mentioned above, the DC did not differ significantly between the 60 and 80 °C groups despite the cytotoxicity being lower in the latter group, and significant differences in cytotoxicity were found between all postcuring temperatures. Park et al. investigated the influence of different dental resin materials on fibroblasts, and found that 3D printed resin specimens showed better biocompatibility than self-cured resin specimens [43]. The production of 3D printed resin does not involve a chemical reaction, and so there is likely to be less cytotoxicity, whereas self-curing technology does involve chemical reactions [43].

Fibroblasts are the most abundant cell type in connective tissues and they have multiple functions and create complex cell signaling networks through their contacts with other cells [44]. Additionally, gingival fibroblasts play essential roles in wound healing, exhibit diversely responses to growth factors, and produce specific extracellular matrix proteins [45]. The present CLSM images revealed clearly differences on the morphology, size, and number of cells with the postcuring temperature. Well-developed morphology and increased cell adhesion were observed in groups that were cured at high postcuring temperatures such as 60 and 80 °C, whereas cells on the specimens postcured at 40 °C demonstrated poor morphology. Substances leached from resin-based dental restorative materials potentially exert adverse effects on basic cellular functions such as proliferation and metabolism, as well as on cell morphology and membrane integrity [46]. Hence, setting the optimal postcuring process is a challenging but crucial step to fulfilling the biological requirement of 3D printed resin since it influences cytocompatibility. Our results suggest that a higher postcuring temperature yields improved biocompatibility of the 3D printed resin.

3D printed resin is increasingly being used in clinical practice due to its satisfactory biocompatibility and mechanical strength, and simple processing. More plaque tends to accumulate on resin materials in the intraoral cavity than on other restorative materials [47]. It is desirable that 3D printed resin prostheses accumulate less biofilm when they are used in long-term applications such as dentures, temporary crowns, and bridges. The present study found that the postcuring temperature significantly affects protein adsorption, with less protein being adsorbed on the surface of the 3D printed resin specimen when using a higher postcuring temperature. However, no significant difference was found in the specimens postcured for more than 30 min. We additionally observed the adhesive characteristics on the surface of the specimens that were postcured at low temperature or that had not undergone a postcuring process (i.e., GS). Previous studies found incomplete polymerization of the resin surface to be associated with atmospheric oxygen in the curing process causing a sticky, uncured oxygen-inhibited layer [48,49]. Moreover, in the presence of monomers, unpolymerized compounds can leach out, which may result in the accumulation of biofilm [50,51]. The present findings indicate that postcuring at 80 °C may be the most favorable postcuring condition and thus result in less biofilm formation. It is plausible that there is an optimal postcuring temperature for enhancing the mechanical properties and biocompatibility of the resin. A favorable postcuring temperature results in resin being polymerized more efficiently by improving the molecular mobility of free radicals and other reactive species [33].

The limitations of the current study included the use of only one type of 3D printed resin material and one UV-light curing chamber. Using different curing chambers and 3D printed materials may produce different outcomes. Future evaluations of the DC on the cross-sectioned surface of printed resin and the two-dimensional mapping of the DC may also provide further insight. In addition, this study only focused on mechanical and biological properties, Vickers hardness and it is possible that the accuracy of the finally produced 3D printed parts would differ with the postcuring parameters. In particular, the accuracy of small and thin 3D printed parts might be reduced at higher postcuring temperature, which should be carefully investigated in future studies. Investigating monomer elution might be useful for thoroughly interpreting the cytotoxic effects of 3D printed resin. Furthermore, the effects of using various postcuring chambers on the properties of 3D printed resin material should be determined in order to better understand the postcuring process.

## 5. Conclusions

This study investigated the effects of the postcuring temperature and time on the mechanical properties, Vickers hardness, biocompatibility, and DC of 3D printed resin. Within the limitations of this study, the following conclusions can be drawn: (1) increasing the postcuring temperature results in significant enhancements of the flexural properties, Vickers hardness, biocompatibility, and protein adsorption; (2) increasing the postcuring time improves the flexural properties, Vickers hardness, and biocompatibility; and (3) a longer postcuring time at a low temperature produces results similar to using a shorter postcuring time at a higher postcuring temperature. 

## Data Availability

The data presented in this study are available on request from the corresponding author.

## Supplementary Materials

The followings are available online at https://www.mdpi.com/article/10.3390/polym13081180/s1, Table S1. Degree of conversion of three-dimensional printed resin under various curing conditions. Data are mean ± standard deviation values. Different uppercase letters indicate a significant difference in postcuring time for the respective postcuring-temperature group in the same row. Different lowercase letters indicate a significant difference in postcuring temperature according to postcuring time. Table S2. HGFs were seeded into 48-well plates at a density of 5 × 10^4^ cells/specimen. The number of cells attached on each specimen (n = 3) was counted after 24, 48 and 72 h of incubation. Mean values are presented. Figure S1: Disk-shaped specimen, with a diameter of 9 mm and a thickness of 2 mm, were designed using a 3D modeling software for Vickers hardness, biocompatibility test and degree of conversion. Figures S2–S4: CLSM images of HGFs cultured (1 × 10^5^ cells/mL) on 3D printed specimens postcured at various temperatures and times after 24, 48 and 72 h of incubation, respectively.
