# Supplementary material for: Effects of Postcuring Temperature on the Mechanical Properties and Biocompatibility of Three-Dimensional Printed Dental Resin Material
Source: Polymers (Basel). 2021 Apr 7;13(8):1180. doi: 10.3390/polym13081180 (PMC8067535; doi:10.3390/polym13081180)

Table S1. Degree of conversion of three-dimensional printed resin under various curing conditions. Data are mean±standard deviation values. Different uppercase letters indicate a significant difference in postcuring time for the respective curing temperature group in the same row. Different lowercase letters indicate a significant difference in postcuring temperature according to postcuring time.

	Green state	15 min	30 min	60 min	90 min	120 min
**40°C**	43.03±1.46 ^{A,a}	58.08±1.28 ^{B,a}	59.63±0.37 $^{C, a}$	60.41±0.78 $^{C, a}$	59.70±0.90 $^{C, a}$	59.37±1.03 $^{BC, a}$
**60°C**	43.03±1.46 ^{A,a}	60.05±0.69 $^{B, b}$	62.01±0.66 $^{C, b}$	62.32±0.52 $^{C, b}$	62.37±0.39 $^{C, b}$	63.02±0.42 $^{C, b}$
**80°C**	43.03±1.46 ^{A,a}	61.38±0.82 $^{B, c}$	61.30±0.67 $^{B, c}$	61.46±0.51 $^{B, c}$	62.73±0.87 $^{C, b}$	62.67±0.29 $^{C, b}$

Figure S1. Disk-shaped specimen, with a diameter of 9 mm and a thickness of 2 mm, were designed using a 3D modeling software for Vickers hardness, biocompatibility test and degree of conversion.

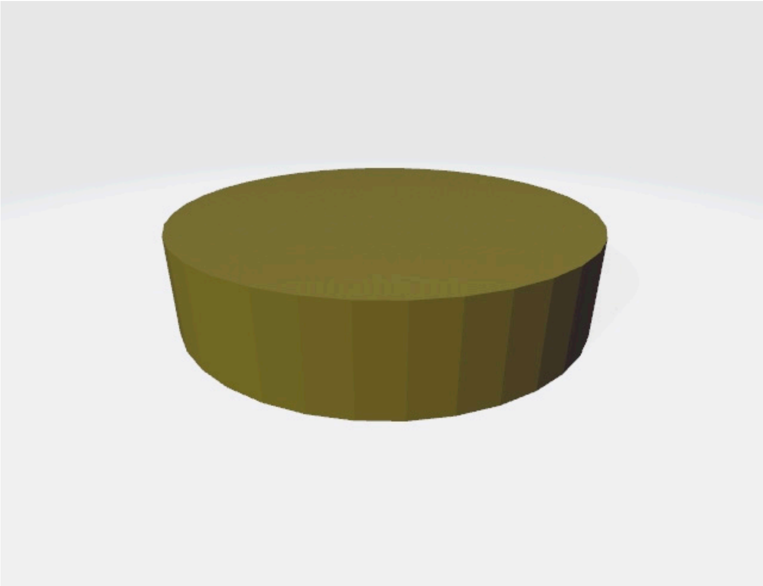

Table S2. HGFs were seeded into 48-well plates at a density of 5×10^4 cells/specimen. The number of cells attached on each specimen (n=3) was counted after 24, 48 and 72 h of incubation. Mean values are presented.

	Number of cells (×10^4 cells)		
	**24 h**	**48 h**	**72 h**
**PC**	3.240	3.333	4.160
**Green state**	0.007	0.020	0
**40°C 15 min**	0.253	0.027	0
**40°C 30 min**	0.233	0.073	0
**40°C 60 min**	0.147	0.013	0
**40°C 90 min**	0.073	0.073	0
**40°C 120 min**	0.540	0.087	0
**60°C 15 min**	0.247	0.053	0.007
**60°C 30 min**	1.033	0.040	0
**60°C 60 min**	0.533	0.133	0.013
**60°C 90 min**	0.760	0.040	0.020
**60°C 120 min**	0.667	0.033	0.013
**80°C 15 min**	1.793	1.633	1.780
**80°C 30 min**	2.233	1.460	1.920
**80°C 60 min**	1.740	1.573	1.880
**80°C 90 min**	2.613	1.420	1.640
**80°C 120 min**	2.1	1.847	2.053

Figure S2. CLSM images of HGFs cultured (1×10^5 cells/ml) on 3D printed specimens postcured at various temperatures and times after 24 h of incubation. (**A**) GS, (**B**) Positive control (**C**) 40°C for 15 min, (**D**) 40°C for 30 min, (**E**) 40°C for 60 min, (**F**) 40°C for 90 min, (**G**) 40°C for 120 min, (**H**) 60°C for 15 min, (**I**) 60°C for 30 min, (**J**) 60°C for 60 min, (**K**) 60°C for 90 min, (**L**) 60°C for 120 min, (**M**) 80°C for 15 min, (**N**) 80°C for 30 min, (**O**) 80°C for 60 min, (**P**) 80°C for 90 min, and (**Q**) 80°C for 120 min.

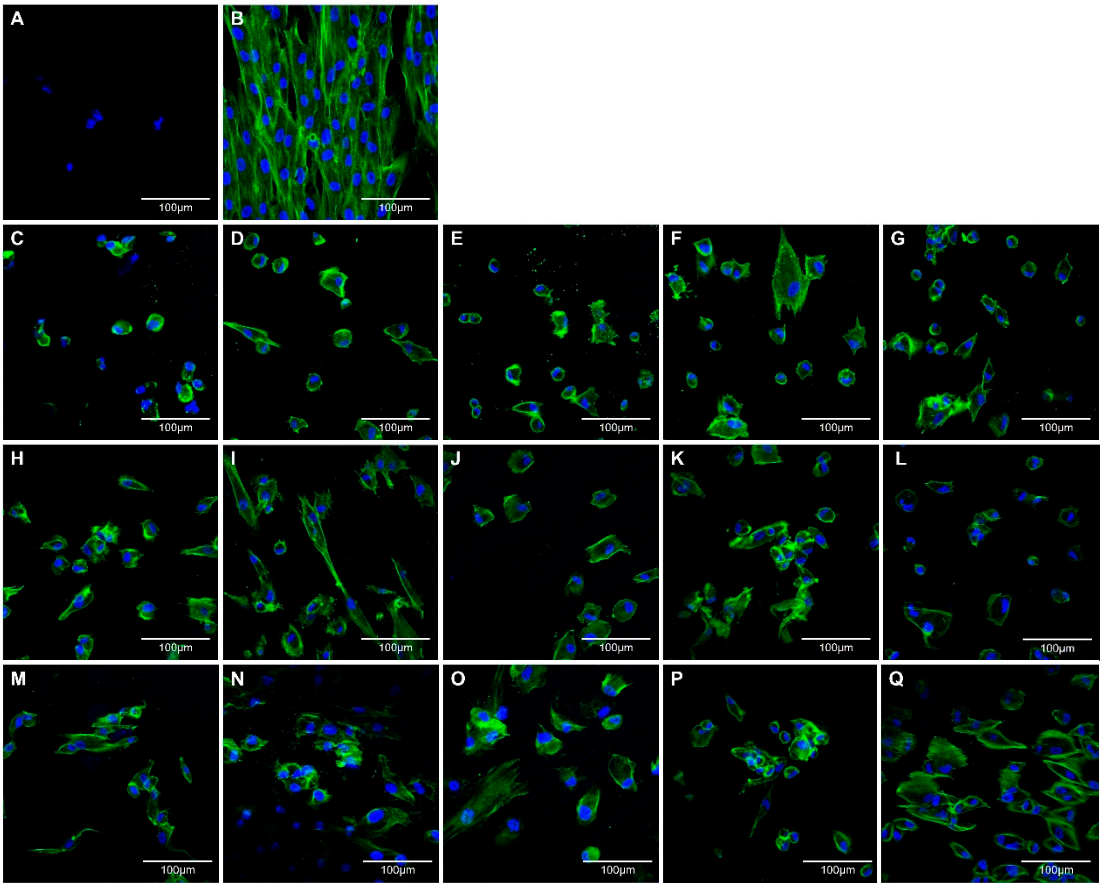

Figure S3. CLSM images of HGFs cultured (1×10[5] cells/ml) on 3D printed specimens postcured at various temperatures and times after 48 h of incubation. (**A**) GS, (**B**) Positive control (**C**) 40°C for 15 min, (**D**) 40°C for 30 min, (**E**) 40°C for 60 min, (**F**) 40°C for 90 min, (**G**) 40°C for 120 min, (**H**) 60°C for 15 min, (**I**) 60°C for 30 min, (**J**) 60°C for 60 min, (**K**) 60°C for 90 min, (**L**) 60°C for 120 min, (**M**) 80°C for 15 min, (**N**) 80°C for 30 min, (**O**) 80°C for 60 min, (**P**) 80°C for 90 min, and (**Q**) 80°C for 120 min.

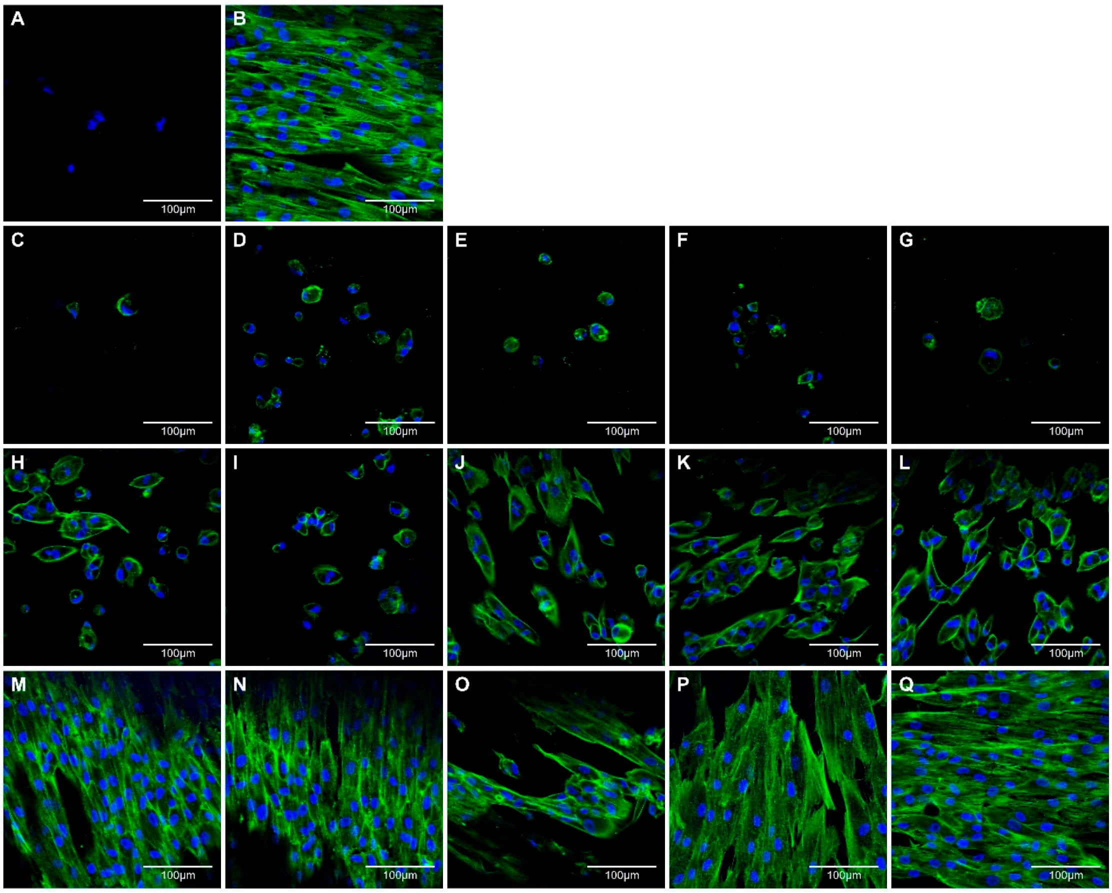

Figure S4. CLSM images of HGFs cultured (1×10^5 cells/ml) on 3D printed specimens postcured at various temperatures and times after 72 h of incubation. (**A**) GS, (**B**) Positive control (**C**) 40°C for 15 min, (**D**) 40°C for 30 min, (**E**) 40°C for 60 min, (**F**) 40°C for 90 min, (**G**) 40°C for 120 min, (**H**) 60°C for 15 min, (**I**) 60°C for 30 min, (**J**) 60°C for 60 min, (**K**) 60°C for 90 min, (**L**) 60°C for 120 min, (**M**) 80°C for 15 min, (**N**) 80°C for 30 min, (**O**) 80°C for 60 min, (**P**) 80°C for 90 min, and (**Q**) 80°C for 120 min.

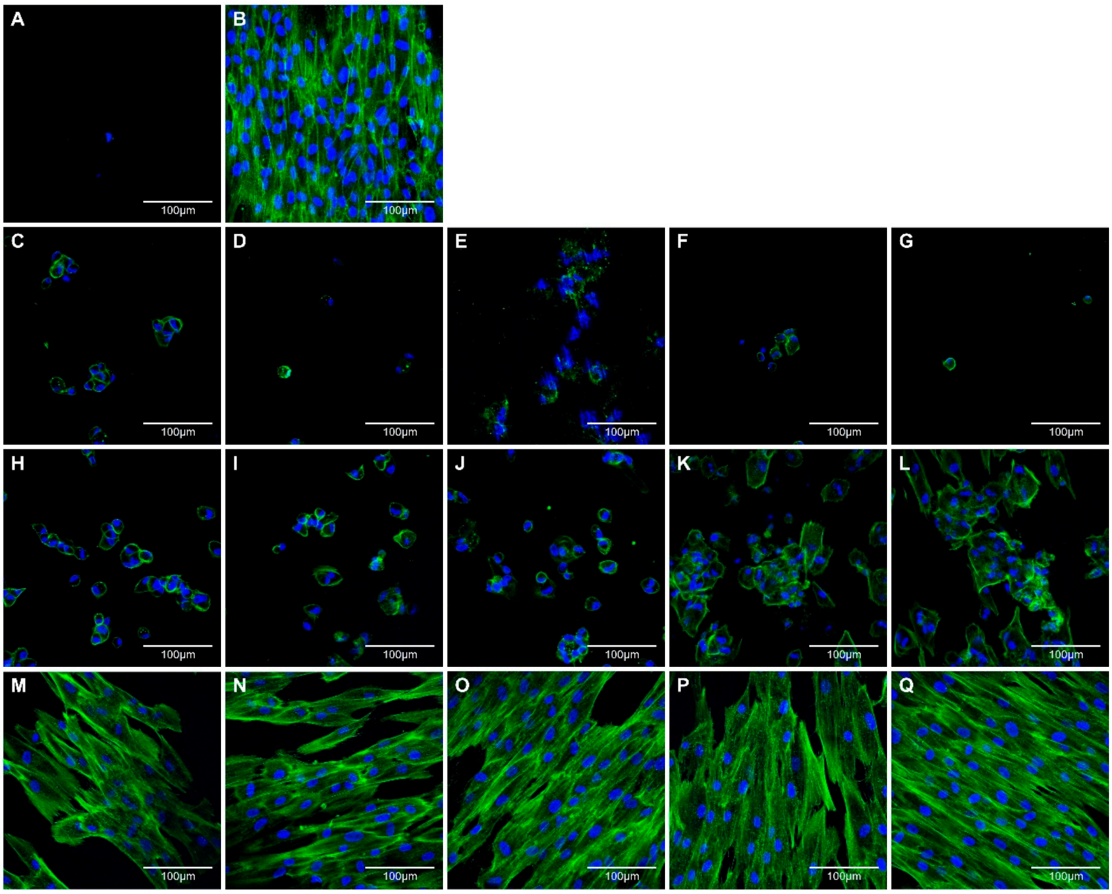

Supplement: Supplementary file 1 [file polymers-13-01180-s001.pdf]
